# Treatment of a Compression Fracture at a Previously Instrumented Lumbar Vertebral Body Using Balloon Kyphoplasty: A Technical Case Report

**DOI:** 10.7759/cureus.4018

**Published:** 2019-02-05

**Authors:** Chad Claus, Evan Lytle, Lucas Garmo, Doris Tong, Boyd Richards

**Affiliations:** 1 Neurosurgery, Ascension Providence Hospital, Michigan State University, College of Human Medicine, Southfield, USA

**Keywords:** compression fracture, instrumented fusion, kyphoplasty, lumbar fusion, lumbar

## Abstract

Compression fractures are common among osteoporotic patients and can be a significant source of pain and disability. Patients who suffer a compression fracture at an instrumented level of a lumbar fusion are most often treated conservatively. Herein, we demonstrate a safe and effective treatment for a vertebral compression fracture (VCF) at a previous level with instrumented fusion and pedicle screw fixation. An 89-year-old female with a history of multiple osteoporotic compression fractures treated with previous kyphoplasties and a prior instrumented fusion at L4-L5 presented with debilitating lower back pain for one week. After failing conservative management, computed tomography (CT) and magnetic resonance imaging (MRI) study of the lumbar spine revealed an acute VCF of the previously instrumented L5 vertebral body. Under biplanar fluoroscopy, a balloon kyphoplasty was performed at the L5 vertebrae utilizing the Inflatable Vertebral Augmentation System (IVAS) from Stryker® (Kalamazoo, MI, USA). We were able to demonstrate that the treatment of an acute VCF with balloon kyphoplasty is feasible in patients who have a history of previous instrumentation with pedicle screws remaining at the fracture level.

## Introduction

As many as 700,000 vertebral compression fractures (VCFs) occur each year in North America, with approximately one-fifth occurring in individuals over the age of 70 [1-2]. Posterior lumbar instrumented fusion is a common procedure in the treatment of degenerative spine disorders. Complications secondary to instrumented fusions include adjacent-segment breakdown, pseudarthrosis, as well as compression fractures [3-8]. However, few reports document treatment options for VCFs in previously instrumented segments [4-5,9] even though they are among the most common fractures of the spinal column due to osteoporosis [1,8]. These types of fractures not only cause debilitating pain, but can often result in disruption of activities of daily living as well as a significant decrease in quality of life [10-11]. Many patients respond favorably to conservative treatments such as bed rest, analgesics, and bracing [1,10]. However, there is evidence that minimally-invasive surgical options such as balloon kyphoplasty performed for patients without significant vertebral instability or neurological compromise can yield faster clinical improvement in pain and mobility, as well as restoration of vertebral body height [12] and a correction of sagittal imbalance [1,8,10,13]. We describe the kyphoplasty technique we used and its outcome on a patient with a previous L4-L5 instrumented fusion, who presented with an acute L5 compression fracture.

## Case presentation

**Figure 1:**
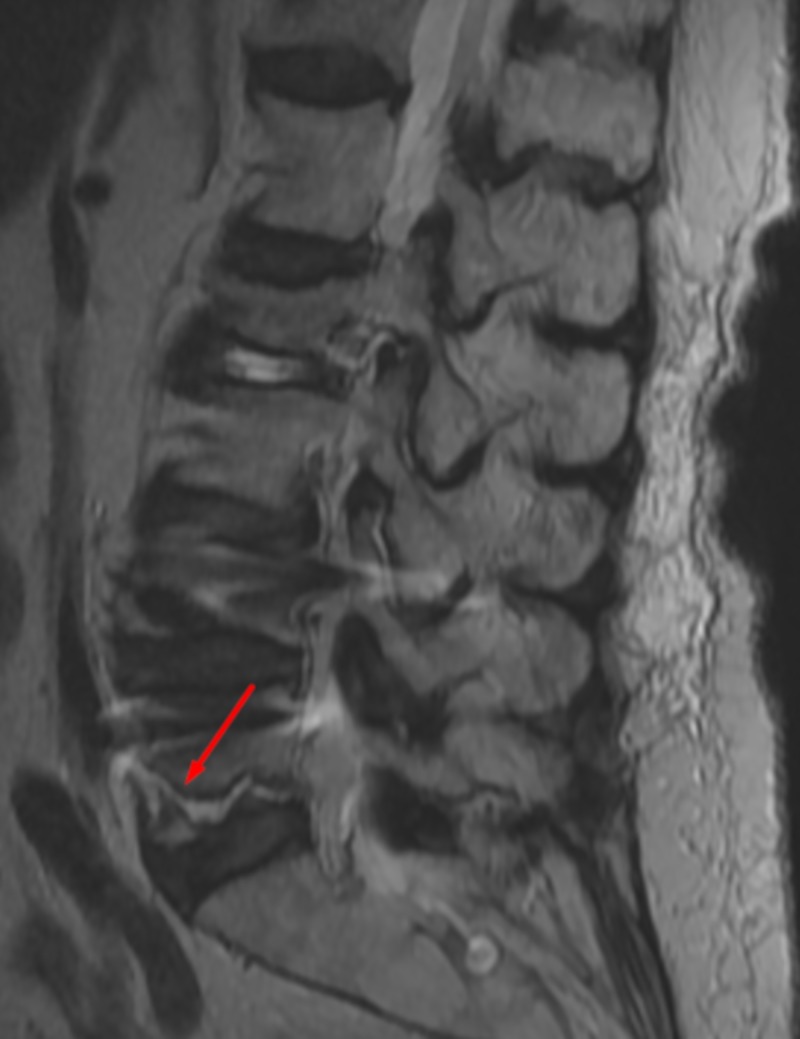
Sagittal Lumbar MRI Sagittal lumbar magnetic resonance imaging (MRI) displaying fracture with associated edema on L5. Arrow indicates fracture.

**Figure 2:**
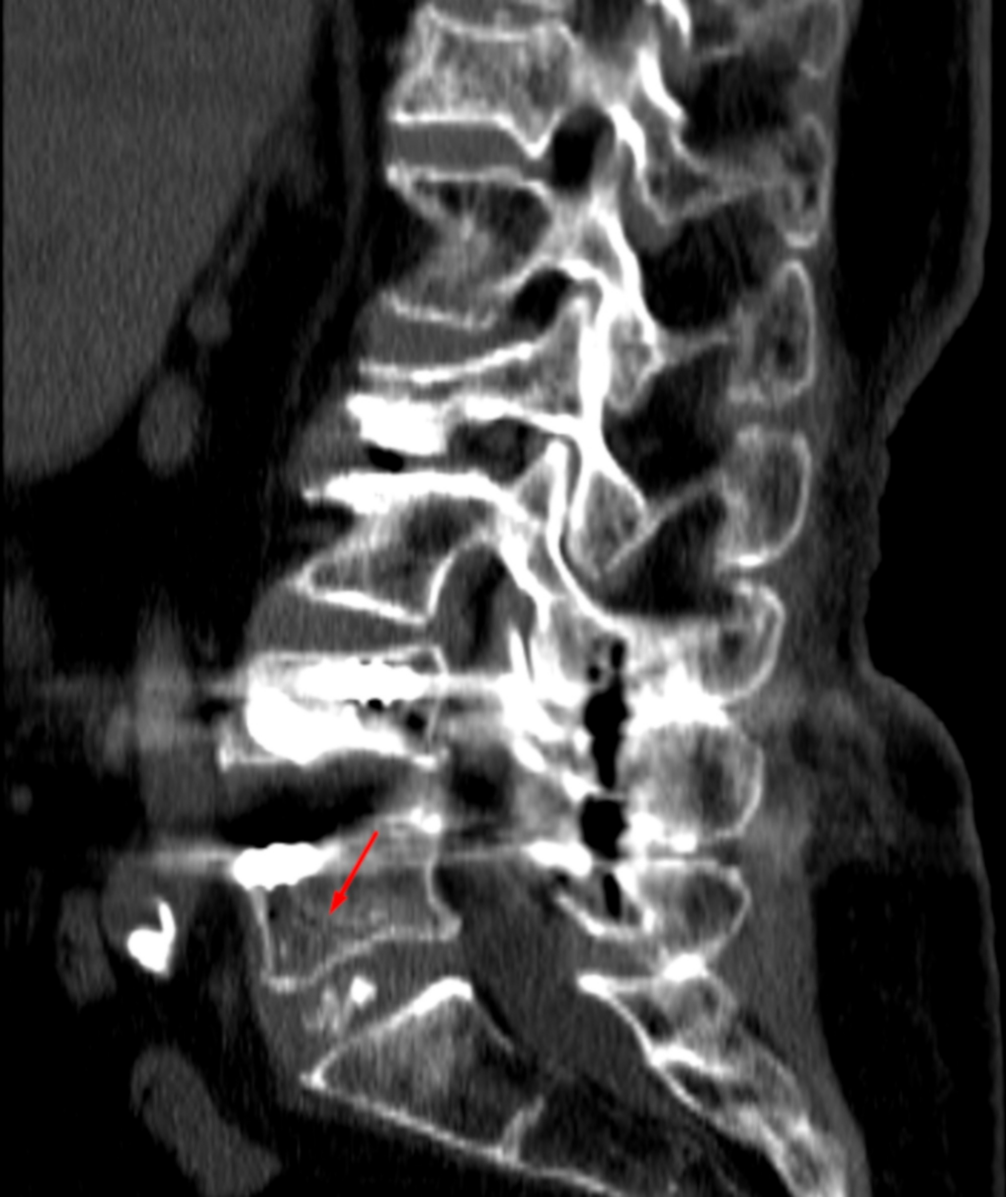
Sagittal CT Lumbar Spine Sagittal computed tomography (CT) lumbar spine revealing fracture line extending superior to and involving the inferior endplate of L5. Arrow indicates fracture.

An 89-year-old female presented with severe, worsening lower back pain for one week. She had a history of severe osteoporosis with previous kyphoplasties at T6, T7, T12, L2, and L4. Four years earlier, the patient underwent an L4-L5 instrumented fusion. An L4 kyphoplasty was performed at the time of the initial L4-L5 fusion. Upon evaluation, there was no neurological deficit. The patient could ambulate with a walker but was severely limited due to pain. Radiographic evaluation revealed a fracture involving the inferior endplate of L5 with associated edema on magnetic resonance imaging (MRI) and computed tomography (CT) (Figures 1-2). Her preoperative visual analogue scale (VAS) in regard to pain intensity was ranked between five to seven on a scale of ten with analgesic medication. After failing conservative treatment with analgesics, and demonstrating almost no tolerance for ambulation, the patient was offered a kyphoplasty and informed consent was obtained. Post-anesthesia care unit (PACU) evaluation after the procedure revealed almost complete resolution of lower back and leg pain. Postoperative VAS evaluation occurred on operative day and revealed a score of two out of ten. The patient was discharged to a subacute rehabilitation facility on the day after the kyphoplasty. On discharge, the patient ambulated with only minimal walker assistance. At a two-month postoperative evaluation, the patient demonstrated continual improvement in ambulation with near complete resolution of her lower back pain.

Surgical technique

**Figure 3:**
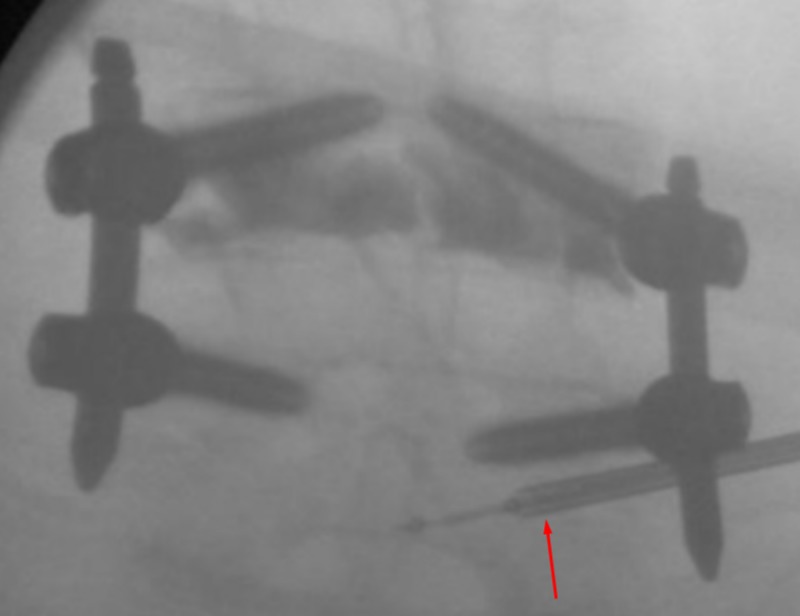
AP View - Insertion Anterior-posterior (AP) fluoroscopic views showing insertion of the osteo-introducer and balloon into the right pedicle inferior and slightly lateral to the screw in the right L5 pedicle. Arrow indicates the osteo-introducer tool.

**Figure 4:**
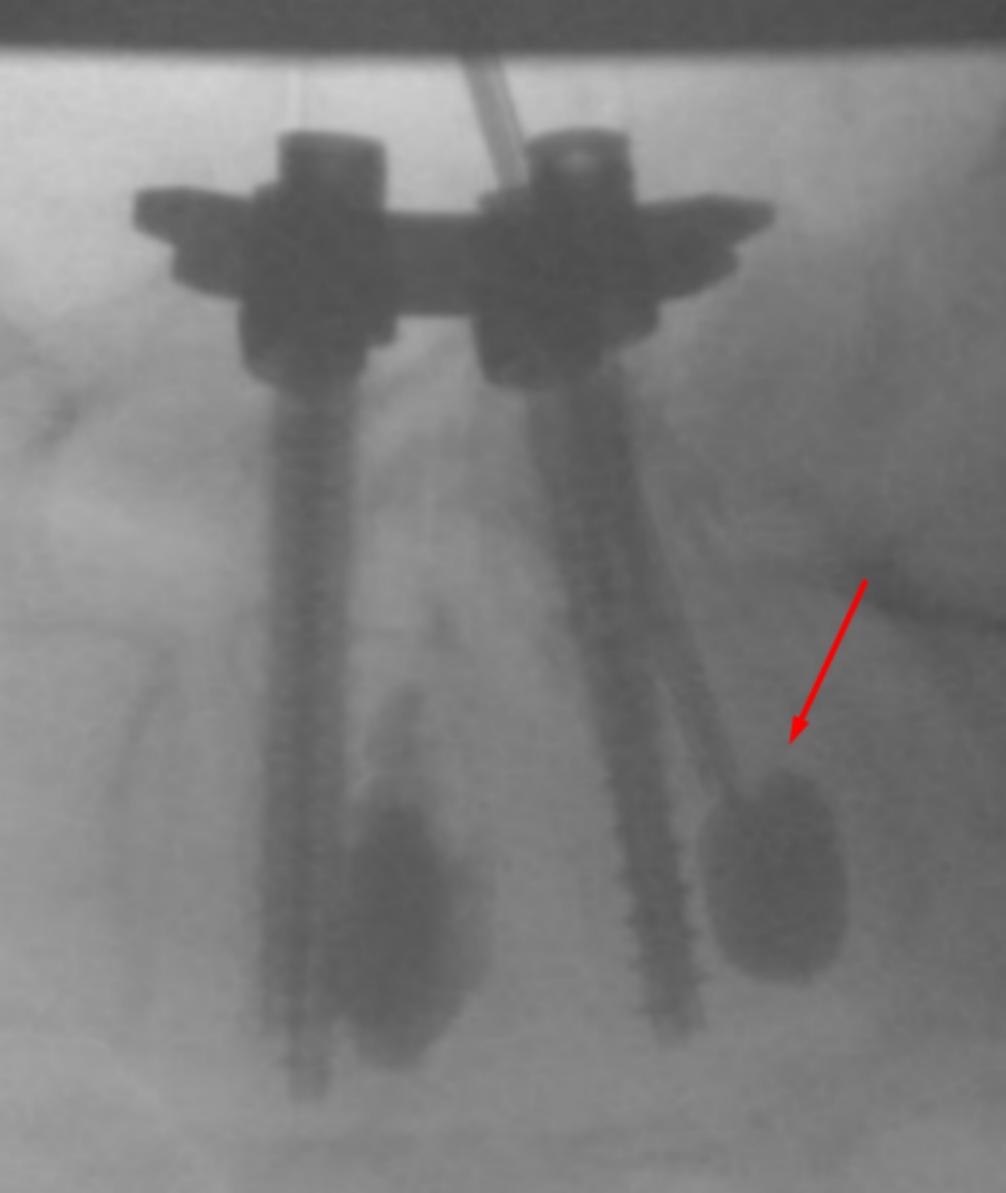
Lateral View - Insertion Lateral fluoroscopic views showing insertion of the osteo-introducer and balloon into the right pedicle inferior and slightly lateral to the screw in the right L5 pedicle. Arrow indicates the osteo-introducer tool.

**Figure 5:**
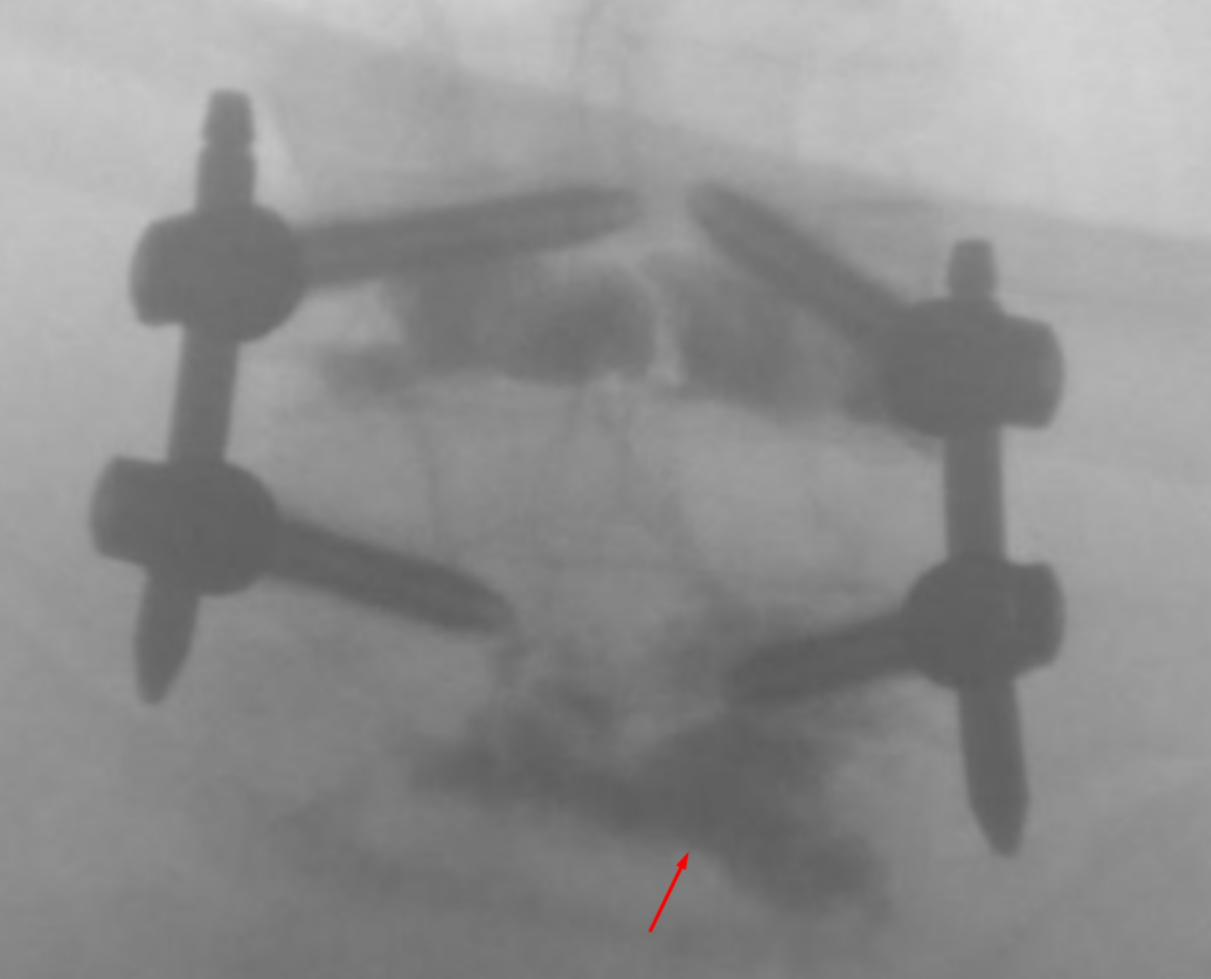
AP View - Fixation Final anterior-posterior (AP) fluoroscopic X-rays demonstrating final fixation of the bone cement. Arrow indicates the bone cement.

**Figure 6:**
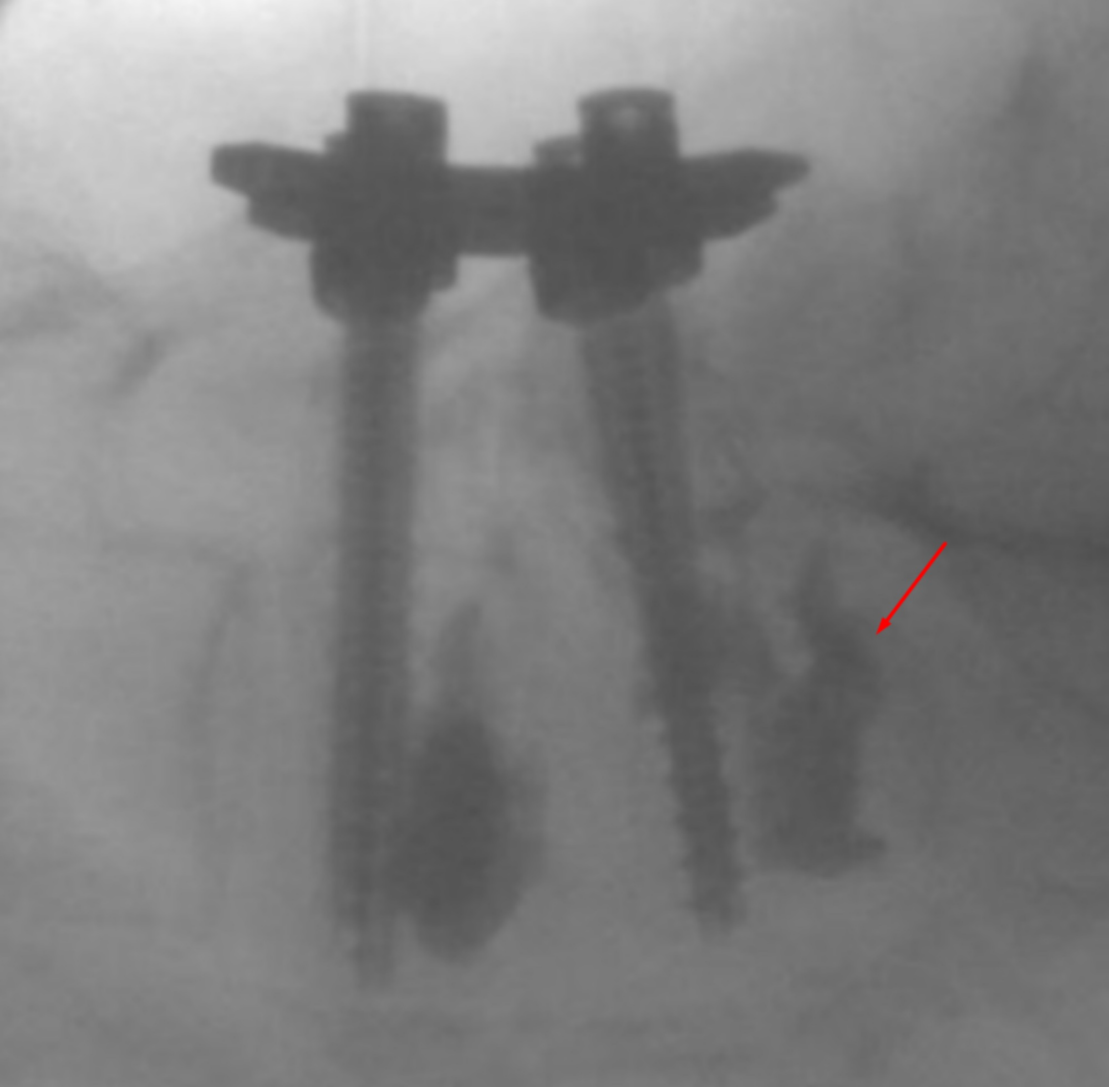
Lateral View - Fixation Final lateral fluoroscopic X-rays demonstrating final fixation of the bone cement. Arrow indicates the bone cement.

The patient was positioned prone on a biplane table. Biplanar fluoroscopy was brought into the field and the targeted vertebral body was localized in both anterior-posterior (AP) and lateral projections. After infiltrating the skin and soft tissue with local anesthetic, a stab incision was made over the right pedicle. The kyphoplasty was performed using the Inflatable Vertebral Augmentation System (IVAS) developed by Stryker® (Kalamazoo, MI, USA). A Jamshidi needle was introduced from a transpedicular approach into the body of L5 via the right pedicle, inferior and lateral to the pedicle screw under biplanar fluoroscopic guidance. The osteo-introducer was then introduced into the vertebral body (Figures 3-4). This created the tract for the inflatable balloon. The balloon was then inserted and inflated to create a cavity within the vertebral body. After the balloon was removed, 5 milliliters of polymethylmethacrylate (PMMA) cement (high viscosity radiopaque bone cement, Stryker®) was infused into the vertebral body. There was no noted extravasation of cement beyond the border of the vertebral body. Once the cement had cured, the Jamshidi needle was removed. Final intraoperative X-rays were performed with fluoroscopy in both AP and lateral projections, confirming acceptable cement fixation without any significant breaching of any borders (Figures 5-6).

## Discussion

VCF can occur in up to 15% of patients who have undergone instrumented spinal fusion [14-16]. The mechanism is likely related to a decrease in bone mineral density and changing biomechanics that follow fusions [17]. This can be increasingly problematic, especially in aging populations. While VCF typically occurs in adjacent segments of previously fused levels, it can also occur in previously fused levels due to osteoporosis [14-16]. The latter presents a unique challenge for treatment, as transpedicular access to the vertebral body is compromised due to the presence of pedicle screws. These patients tend to get offered conservative treatment only and suffer from severe pain and associated morbidities due to prolonged bed rest and decreased activity. The recovery period with conservative treatment of analgesics, physical therapy, and bracing could last up to eight to twelve weeks [18]. This case demonstrates that despite the presence of pedicle screws, the vertebral body in the lumbar spine can still be accessed through careful fluoroscopic guidance using biplanar fluoroscopy. Most pedicles in the lumbar spine are between 8 and 15 millimeters in diameter, depending on the level, while most pedicle screws have an outer diameter of 5 to 7 millimeters [19]. This difference can still allow ample space to access the vertebral body. The screw itself can serve as a point of reference to guide the Jamshidi needle into the pedicle. Newer surgical instruments such as curved needles and directional Jamshidi needles facilitate better crossing of the midline using a uni-pedicular approach, which can accommodate unilateral treatment success and reduce the risk of compromising both pedicles. The outcome of this case demonstrates the described technique to be a safe and effective minimally invasive surgical alternative to conservative treatment. The kyphoplasty procedure in this patient led to an expedited recovery when compared to conservative treatment only.

## Conclusions

This technical case report demonstrates that the treatment of acute lumbar VCF with balloon kyphoplasty is feasible even in the presence of pedicle screws at the affected vertebrae. Further randomized study will be needed to evaluate the superiority of this procedure over other treatment options.
